# Accidental ingestion of coins by children: management of the ENT Department of João XXIII Hospital

**DOI:** 10.1016/S1808-8694(15)30992-7

**Published:** 2015-10-19

**Authors:** Cheng T-Ping, Cassimiro Afonso Nunes, Gabriel Rabelo Guimarães, João Penna Martins Vieira, Luc Louis Maurice Weckx, Tanner José Arantes Borges

**Affiliations:** a MS in Otorhinolaryngology - CCP - UNIFESP-EPM. Attending ENT - João XXIII Hospital; b Otorhinolaryngologist, Head of the Otorhinolaryngology Department - João XXIII Hospital; c Head and Neck Surgeon João XXIII and Felício Rocho Hospitals; d Attending ENT - João XXIII Hospital; e Full Professor - Paulista School of Medicine - UNIFESP, Head of the Post Graduation Program in Otorhinolaryngology and Head and Neck Surgery - Paulista School of Medicine - UNIFESP; f Attending ENT - João XXIII and Felício Rocho Hospitals. Hospital João XXIII, Belo Horizonte, MG

**Keywords:** foreign body, children, cricopharynx, esophagoscopy, esophagus, coins

## Abstract

The ingestion of foreign bodies by children is frequently seen in emergency departments. ENTs can manage those lodged in the esophagus but experience is important for a successful intervention.

**Aim:**

Describe seven cases of children that ingested coins, managed at the ENT Department of João XXIII Hospital.

**Study design:**

Clinical/prospective.

**Materials and Methods:**

We describe seven cases (gender, age, family status, coin size and treatment/evolution).

**Results:**

Age ranged from one to nine years. Two patients were only children and five were the youngest in their families. Coins sizes ranged from 1.9 to 2.5 cm. After eight hours of observation, three cases were treated in the surgery room because the foreign body was lodged in the cricopharynx. Four cases resolved spontaneously.

**Conclusion:**

The ENT department has good results removing coins lodged in the upper esophagus using forceps and laryngoscopy; and also using rigid esophagoscopy for the lower esophagus. In this study it was not possible to establish the importance of coin size and patient age in attempting to predict spontaneous resolution, nor if the child being an only child or the youngest in the family may have some predisposition in this kind of accident.

## INTRODUCTION

Cases of accidental foreign body ingestion by children are frequent in emergency rooms. The otorhinolaryngologist is the most qualified professional to manage ear, nose and oropharynx foreign bodies, and he/she may also manage the ones located in the esophagus. Coins, because of their shape and ease of reach, are the foreign bodies most frequently found in the latter^1^, ^2^, ^3^. These patients have to be carefully assessed and if the coin remains lodged in the esophagus after 8 hours of fasting - time necessary for a safer procedure - the foreign body should be removed under general anesthesia in order to avoid complications^3^. Besides patients and families’ stress, especially considering the former's age and the difficulties brought about by the minute anatomy of their respiratory tract, the removal can be even more dangerous than the foreign body itself. To avoid complications, experience and skill acquired in specific training, together with the help from other specialists such as pediatricians, general and thoracic; and the anesthesiology team, all most needed when complications arise.

## OBJECTIVES

Describe patient care and the evolution of seven children who were sequentially seen at the emergency ENT department at the Hospital João XXIII, in Belo Horizonte - MG, after having ingested a coin. We considered coin size, patient's age and the removal method for this type of foreign body in our institution.

## MATERIALS AND METHODS

We assessed seven sequential cases of children who swallowed coins and were seen from June 15th, 2004 through August 18th, 2004, during the day shift (7am through 7pm) on Wednesdays, at the ENT Department of the Hospital João XXIII (Public State Emergency Hospital for polytraumatized patients and clinical/surgical emergency cases from different specialties), located downtown Belo Horizonte - MG- Brazil.

Care is based on a detailed clinical history, physical exam, simple neck-chest-abdominal antero-posterior-view X-Rays. It is of the utmost importance to establish the probable time of ingestion and that of the last meal, as well as past history of esophagus diseases and diseases that may increase the risk of a procedure under general anesthesia, if needed be. If the coin is seen in the gastrointestinal track at the initial X-Ray evaluation, the patient is discharged; however, if the foreign body is still located in the esophagus, the patient should remain under observation until 8 hours of fasting have passed.

If the X-Ray taken after 8 hours of fasting shows the foreign body in the gastrointestinal tract, the patient is discharged and instructed to look and see if the coin comes out in the feces, and he/she should return to the hospital if the coin is not seen, or if any complication arise, for instance -abdominal pain. If the coin remains in the esophagus, the patient is then taken to the surgery center for removal under general anesthesia.

**Figure 1:**
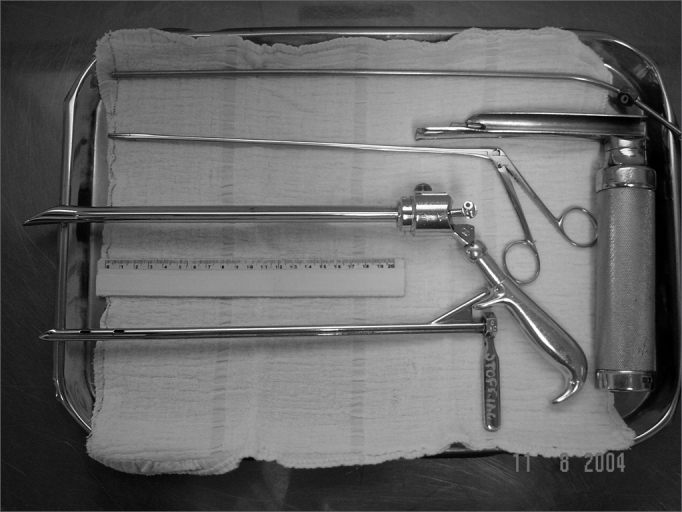
Instruments used: aspirator, straight laryngoscope, apprehension forceps and rigid esophagoscopes.

When the coin remains lodged in the esophagus upper third, the patient is anesthetized and monitored without orotracheal intubation in order to undergo extraction through a straight laryngoscope and long forceps. If that is not possible, the patient is intubated and rigid esophagoscopes may be necessary (Figure 1). After coin removal in the surgical center, the patient remains under observation for up to four hours and, provided there are no complications, the patient is discharged.

The coins were measured with a ruler. The size of the coins that spontaneously passed through to the gastrointestinal tract was estimated and measured by comparison with coins of similar monetary value, according to the description given by the patients’ relatives.

## RESULTS

Patients were sequentially ordered in Table 1, including age, gender, status in the family, coin size, approach used and evolution.

**Table 1:** Distribution of patients in sequential order of their care.

Cases	Gender	Age	Status	Coin size	Approach and evolution
1	Masculino	3y and 5m	Youngest child	2,2cm	Intubation and extraction with rigid esophagoscope and forceps
2	Feminino	1y and 7m	Youngest child	1,9cm	Mask ventilation and extraction with straight laryngoscope and forceps
3	Masculino	4y and 7m	Only child	1,9cm	Intubation and extraction with rigid esophagoscope and forceps
4	Feminino	2y and 6m	Youngest child	1,9cm	Spontaneous gastrointestinal descent
5	Masculino	6y and 10m	Youngest child	2,3cm	Spontaneous gastrointestinal descent
6	Feminino	9y and 5m	Only child	2,5cm	Spontaneous gastrointestinal descent
7	Masculino	6y	Youngest child	1.9cm	Spontaneous gastrointestinal descent

These accidents involved seven children with ages varying between one and nine years. The age of those patients in whom the coin remained in the esophagus varied from one year and seven months to four years and seven months, two boys and one girl. The age of the four children in whom the coin descended spontaneously, varied from two years and six months to nine years and five months, two boys and two girls. Of the seven children, two were only children and five were the youngest in the family.

**Figure 2:**
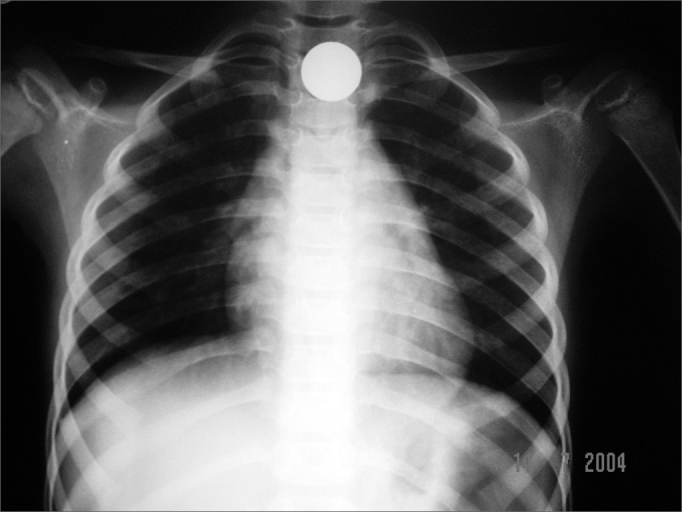
Case 2: 1.9cm coin in the crichopharyngeal area at patient admission.

**Figure 3:**
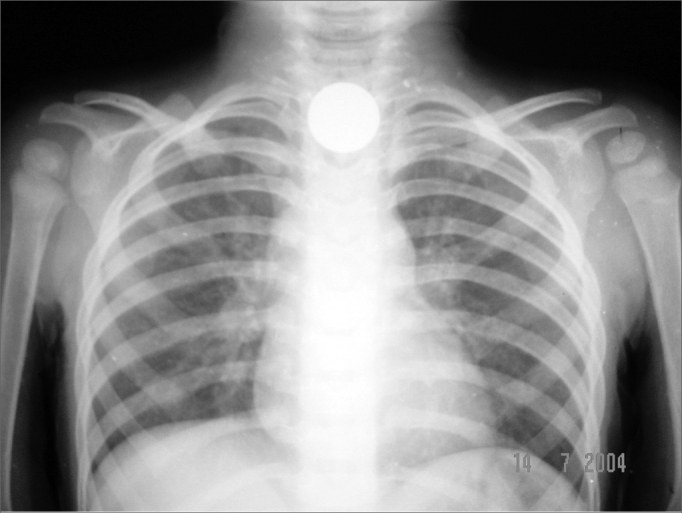
Case 2: 1.9cm coin in the crichopharyngeal area after 8 hours of fasting.

Of the seven foreign body ingestion cases, three (cases 1, 2 and 3) required removal at the surgical center because the X-Ray carried out after eight hours of observation during fasting showed the coin still in the esophagus, at the upper portion of the cricopharynx. Due to the location pinpointed by the radiography just before surgery, the initial procedure was inhaling anesthesia and exploration with straight laryngoscope in a removal attempt without intubation. Cases 1 and 3 required orotracheal intubation and rigid esophagoscope for coin removal, because it had descended beyond the reach of the laryngoscope. In case 2, the coin remained at the cricopharynx region, allowing its removal without the need for orotracheal intubation (Figures 2 and 3).

For cases 4, 5, 6 and 7, the coin spontaneously descended after 8 hours of fasting and observation. The patients were educated and discharged.

**Figure 4:**
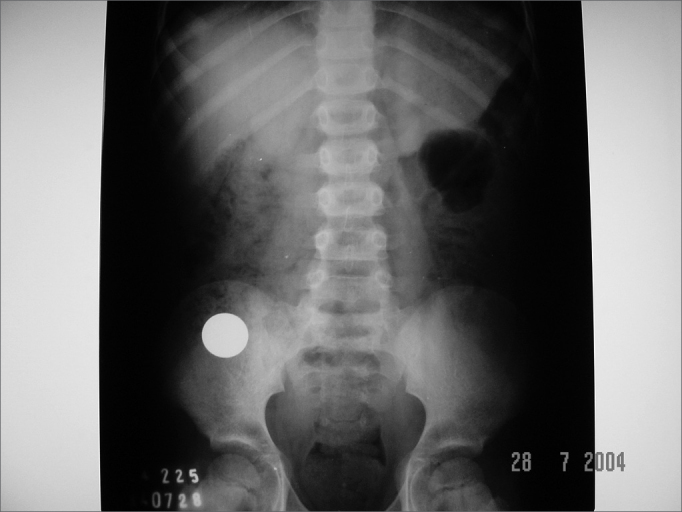
Case 5: 2.3cm coin that spontaneously descended to the gastrointestinal tract.

**Figure 5:**
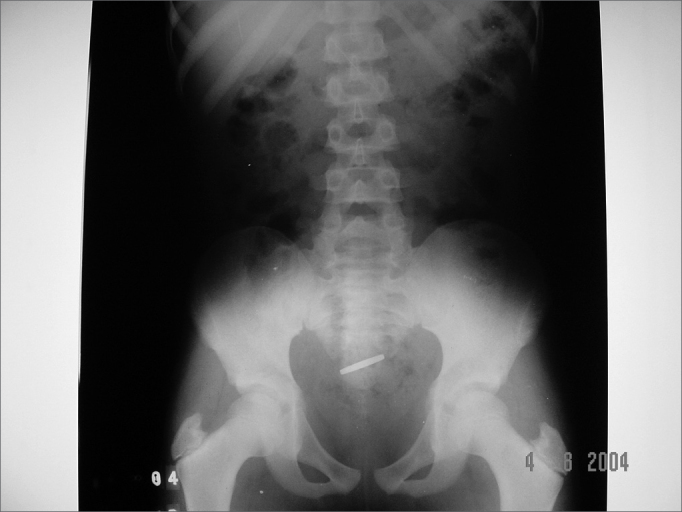
Case 6: 2.5cm coin that spontaneously descended to the gastrointestinal tract.

The coin size varied from 1.9cm to 2.5cm. Cases 2, 3, 4 and 7 were related to 1.9cm coins (cases 2 and 3 evolved with the coin impacted in the esophagus, while cases 4 and 7 resolved spontaneously). Cases 5 and 6 are related to bigger coins (2.3 and 2.5cm in diameter, respectively) and also resolved spontaneously (Figures 4 and 5); however, they were also the eldest children in the group (6 years and 10 months and 9 years and 5 months of age, respectively).

## DISCUSSION

Children commonly ingest foreign bodies, especially coins, thus making it a frequent occurrence in the pediatric emergency and urgency departments^1^. Coins are the most common foreign bodies ingested by children and they usually lodge at the cricopharynx^1^, ^2^, ^3^. Its thin and round shape usually facilitate ingestion, and rarely brings about complications^1^. Complications related to foreign body ingestions are low, however morbidity may be severe, or even put the patient's life in danger^4^.

Younger children are more prone to accidental ingestion of foreign bodies because their dentition is not yet complete, the neuromuscular mechanisms related to swallowing and upper airway protection are not yet fully developed, and moreover, children tend to explore the world around them through their mouths. In older children, cases of esophagus foreign bodies happen more seldom because there is less of a chance they would ingest something inadequate^3^.

### Anatomy of the esophagus

The esophagus is a muscular, thin and vertical tubular organ (it starts in the middle and slowly twists to the left, returning to the middle at the level of the 5th thoracic vertebrae), that goes from the hypopharynx to the stomach. Its is internally lined by stratified epithelial mucosa. It starts in the inferior border of the cricoid cartilage (6th cervical vertebrae), goes through the neck, through the upper mediastinum and ends at the cardia orifice, in the stomach (11th thoracic vertebrae), averaging 23 to 25 cm in length, in adults. In the abdomen it turns to the left and then forward, averaging 1.5 to 3cm in length^5^.

The esophagus presents four narrowing sites: 1) cricopharynx (also known as upper esophagic sphincter, 15cm away from the incisive teeth - where most frequently the foreign bodies are found and where iatrogenic perforations happen more often), 2) aortic cross (located 7cm from the first); 3) left bronchi compression (4cm from the 2nd) and 4) cardia (known as lower esophagic sphincter, located some 40 cm away from the incisive teeth)^5^.

### Management

Management options for coins in the esophagus are: 1) observation, 2) extraction by Foley catheter, guided or not by fluoroscopy, 3) rigid or flexible esophagoscopy, 4) extraction by a Magill forceps, or 5) push the coin to the stomach^2^, ^6^. The method of choice depends on the efficacy, safety and the cost of the procedure^7^.

Asymptomatic patients who go to the emergency room after having ingested a coin with less than twenty hours of evolution have little chances of complication, however if it remains stuck, removal is recomended^7^. Any foreign body, in any portion of the esophagus should not remain there for more than two days, because then we have an increase in the likelihood of complications occurring^4^. Foreign bodies that remain stuck for longer periods, or that cause important local inflammatory reaction increase morbidity^3^.

In 1996 Conners et al. recommended that patients with coins lodged in the distal portion of the esophagus should be observed for 24 hours because these coins can spontaneously migrate to the stomach. Soprano et al. showed that asymptomatic patients have a 28% chance of spontaneously passing a coin to the stomach within a 24 hour period. Coins located in the middle third or distal third of the esophagus have 33% and 37% chance of spontaneously migrating to the stomach, respectively^7^. This study recommends that the asymptomatic cases of coins lodged in the middle and distal thirds may be observed at home for a period of 24 hours, however if they remain in the esophagus they should be removed. Home watch have a better cost-benefit ratio than hospital stay for observation^8^. In cases of coins located at the cricopharynx, watching alone does not bear good results^2^. X-rays should be always done just prior to any procedure, because the coin may descend to the abdomen^5^.

In those cases of coins located at the cricopharynx, Mahahafz starts the procedure using a breathing mask and inhalation, if direct view removal is not possible; he then proceeds to orotracheal intubation and either rigid or flexible esophagoscopy^2^.

The Magill forceps may be used when the coin is lodged in the cricopharynx as an initial procedure or after failure in using the Foley catheter^2^.

In order to avoid esophagus perforations, rigid or flexible endoscopic removal should be carried out by the pediatric surgeon or endoscopy specialist under general anesthesia^3^, ^4^.

When the coin is located at the distal third, knowledge about the anatomy is fundamental for patient safety. In general, one should avoid to blindly push the foreign body towards the stomach^4^. Pushing the coin towards the stomach requires monitoring after the procedure^3^. 80-90% of all foreign bodies that fall in the stomach will spontaneously pass through the digestive tract, however 10-20% will require non-surgical interventions and 1% will require surgery^4^.

Coin extraction through the use of the Foley balloon is a method described over 25 years ago and should be used by a pediatric radiologist, without sedation, and physically restraining the patient if necessary. Such method requires an experienced radiologist in order to make the procedure safe. A 12F or 14F balloon is inserted through the nose, having the patient laying in 15° Trendelenburg decubitus. The cost of esophagoscopy comes to 400 times higher than that of the Foley balloon^7^.

In general, the method chosen depends on procedure efficiency, efficacy, safety and cost, and also the experience of the department^7^.

### Procedure-related Complications

Complications rarely happen, however there may be aspiration and upper airway obstruction, mucosal damage, esophageal erosion and trachea-esophageal fistulas. Complications occur mainly because of invasive removal attempts. 1.8% of the patients who undergo Foley catheter removal attempt present some complications (epistaxis, vomits and transitory respiratory failure)^8^. Esophageal perforations have not been described in the cases in which the Magill forceps was used. The risk of esophageal perforation with the rigid or flexible endoscope varies between 5-10%, although published papers stated that there were no coin-extraction-related perforations in the samples studied^2^.

## FINAL COMMENTS

The cases of esophageal foreign bodies reported by the present study were all coin-related, and they may be managed by the otorhinolaryngologist, as long as he/she has the proper training and the help of other specialists such as endoscopists, pediatricians and general surgeons. Although some papers mention the extraction by the Foley balloon as being the method of choice due to its safety and cost^7^, the Department of Otorhinolaryngology of the João XXIII Hospital has enjoyed good results with the straight laryngoscope and forceps in those foreign bodies located in the cricopharynx; and rigid esophagoscopy for those distally positioned. It is very important to have the support from the anesthesiology team in order to avoid transoperative complications. The initial approach is to confirm the presence of the coin through an x-ray and repeat it after eight hours - time necessary to add safety to the anesthesia and assessment of the patient's general status by the pediatrician if surgery becomes necessary. During this observation period, the patient is preferably kept in the hospital, because often times in these cases the patients come from very unfortunate social and economic situations. The procedure should be carried out by experienced otorhinolaryngologists and the department should count on the support of general and thoracic surgeons in case iatrogenic esophageal perforations should happen.

In this small series of patients, it was not possible to conclude whether or not coin size or patient age may influence the spontaneous passing of the foreign body. It was also not possible to determine if the only children or the youngest ones in the families are more susceptible to this type of accident. In agreement with other authors^3^, we believe prevention and parents’ education considering the children's age and their own characteristics to be fundamental in the prevention of such household accidents, like the accidental ingestion of coins.

## References

[1] Brasin A, Elitsur Y (2004). Esophageal stenosis, a rare complication of coin ingestion: case report. Gastrointest Endosc.

[2] Janik JE, Janik JS (2003). Magill forceps extraction of upper esophageal coins. J Pediatr Surg.

[3] Lay ATY, Chow TL, Lee DTY Kwok SPY (2003). Risk factors predicting the development of complications after foreign body ingestion. Br J of Surg.

[4] Lemberg PS, Darrow DH, Holinger LD (1996). Aerodigestive tract foreign bodies in the older child and adolescent. Ann Otol Laryngol.

[5] Lay ATY, Chow TL, Lee DTY Kwok SPY (2003). Risk factors predicting the development of complications after foreign body ingestion. Br J Surg.

[6] Marques MPC, Couto FD, Fim LA, Nogueirol RB, Oliveira VS (1997). Manipulação do corpo estranho de esôfago: revisão de 5 anos. Rev Bras Otor.

[7] Swischuk LE (2003). Swallowed penny. Pediatr Emerg Car.

[8] Sharieff GQ, Brousseau TJ, Bradshaw JA, Shad JA (2003). Acute esophageal coin ingestions: is immediate removal necessary?. Pediatr Radiol.

[9] Dokler ML, Bradshaw J, Mollitt DL, Tepas JJ (1995). Selective management of pediatric esophageal foreign bodies. Am Surg.

